# HIV infection disclosure, treatment self-efficacy and quality of life in HIV-infected MSM receiving antiretroviral therapy

**DOI:** 10.1186/s12879-022-07932-z

**Published:** 2022-12-13

**Authors:** Wenwen Jia, Kedi Jiao, Jing Ma, Meizhen Liao, Chunmei Wang, Dianmin Kang, Yuxi Lin, Yu Yan, Yijun Li, Chunxiao Cheng, Jing Meng, Lina Wang, Xuan Yang, Yanwen Cao, Zhonghui Zhao, Xinting Wang, Wei Ma

**Affiliations:** 1 Department of Epidemiology, School of Public Health, Cheeloo College of Medicine, Shandong University, 44 West Wenhua Road, Jinan, 250012 Shandong People’s Republic of China; 2 Institution for AIDS/STD Control and Prevention, Shandong Center for Disease Control and Prevention, 16992 Jingshi Road, Jinan, 250014 Shandong People’s Republic of China; 3 Shandong Public Health Clinical Center, 12 East Martyrs Mountain Road, Jinan, 250132 Shandong People’s Republic of China

**Keywords:** HIV/AIDS, Disclosure, Men who have sex with men, Self-efficacy, Quality of life

## Abstract

**Background:**

Research on the relationship between disclosure of HIV status to male sexual partners (HIV disclosure) and quality of life (QOL) revealed complex and even contradictory results. The impact of HIV disclosure on various domains of QOL and the mediation effect between them are unclear. The purposes of this study were to explore the impact of HIV disclosure on QOL among men who have sex with men (MSM), and whether HIV treatment self-efficacy mediated these relationships.

**Methods:**

The data came from a baseline survey on the design of a randomized control trial conducted in Shandong, China. A total of 579 MSM patients were included. SPSS 24.0 was used to conduct independent samples *t test*, one-way analysis of variance and nonparametric tests and the PROCESS macro was used to conduct mediation analysis.

**Results:**

Among 579 participants, 16.06% disclosed their HIV infection status to their male sexual partners. The effect of HIV disclosure on QOL was mediated by treatment self-efficacy. Self-efficacy played partial mediating role in social relationships, meaning that HIV disclosure had both direct and indirect effects on this factor. In the overall QOL and domains of physical, psychological, independence, and environment, HIV disclosure had an indirect effect only through self-efficacy and no significant effect on the spirituality domain.

**Conclusions:**

The results emphasize the importance of HIV disclosure and self-efficacy on the QOL of MSM patients and suggest that health care providers should assist MSM patients in deciding whether to disclose their HIV status during daily medical services.

**Supplementary Information:**

The online version contains supplementary material available at 10.1186/s12879-022-07932-z.

## Background

Acquired immune deficiency syndrome (AIDS) is an infectious disease caused by Human immunodeficiency virus (HIV), which poses great challenges to global public health [1]. Antiretroviral therapy (ART) has shown great efficacy in viral suppression, immune function recovery and HIV/AIDS-related mortality reduction, transforming AIDS from a fatal disease into a manageable chronic disease [2]. With the extension of life expectancy for people living with HIV (PLWH), quality of life (QOL) has become an important topic for PLWH. The Joint United Nations Programme on HIV/AIDS (UNAIDS) considered the QOL to be the ‘fourth 90’ target of HIV/AIDS testing and prevention; that is, 90% of PLWH with viral load suppression remain in a healthy state [3]. In China, the risk of HIV infection among men who have sex with men (MSM) is growing rapidly, and MSM with HIV/AIDS still have many problems in the physical, psychological and social domains [4–6]. Improving the QOL of MSM patients has become one of the primary goals of patients and their medical service providers.

Among MSM patients, disclosure of HIV status to male sexual partners (“HIV disclosure” hereafter) was the first step to establishing a supportive relationship with sexual partners. HIV disclosure also played an important role in HIV prevention, which could minimize stigmatization and discrimination against PLWH [7, 8]. Current studies have indicated that HIV disclosure can reduce the occurrence of high-risk sexual behaviours, such as unprotected anal intercourse (UAI) behaviours and multiple sexual partners, thus decreasing the risk of HIV transmission [9, 10]. A previous review demonstrated that HIV disclosure of MSM could not only reduce the transmission of HIV from patients to negative partners but also decrease the risk of repeated infection due to reinfection with other viruses in MSM patients [11]. Mathematical models showed that disclosure of HIV status to sexual partners can reduce the risk of HIV transmission by approximately 40% [12, 13]. In addition, HIV disclosure was linked to less mortality among HIV-infected individuals on ART and could help MSM patients improve their ART adherence [14]. Good adherence of those who disclosed HIV infection status was found to be 1.6 times that of those who did not [15, 16]. In addition to preventing HIV transmission and promoting treatment adherence, HIV disclosure was also related to many positive results, such as increasing social support, reducing psychological distress, and improving happiness [17–19]. However, MSM faced many challenges in disclosing their HIV serostatus to their male partners. Although HIV disclosure was important for MSM to obtain social support, potential risks such as loss of financial support, breakdown of partnership, abuse or fear of discrimination prevented them from disclosing [20, 21]. There was a difference between regions and countries in terms of HIV disclosure [22, 23].

The relationship between the disclosure of HIV status and QOL revealed complex and even contradictory results [24–26]. For example, some studies found that MSM patients who were hiding their HIV status were related to poor QOL [25, 26], but others showed that disclosure was related to poor physical and mental health [24]. This difference might be related to the various disclosure methods, regions and cultural backgrounds. Although it has been showed that there is a specific correlation between disclosure and QOL, the path of the role of disclosure and the impact on the specific areas of QOL have not been fully explained.

Self-efficacy, as first proposed by Bandura, refers to a person’s confidence in their ability to complete a specific behaviour [27]. According to social cognitive theory (SCT) [27, 28], self-efficacy is a promoting factor to take action and one of the four core determinants of health promotion [27, 28]. People with higher self-efficacy are more likely to engage in health promotion behaviours. Several studies [29, 30] showed that self-efficacy had an important effect on QOL, indicating that improving HIV treatment self-efficacy was essential to promote QOL in patients with HIV/AIDS. PLWH with high self-efficacy might have more optimistic attitudes and more positive coping strategies, resulting in higher QOL scores in various domains [30]. Moreover, some studies found a negative correlation between disclosure and HIV treatment self-efficacy due to a lack of economic, emotional or psychological support and poor coping skills in partners [7, 20]. Some researchers began to explore the impact of self-efficacy as a mediator of the QOL of MSM patients. Although existing studies have demonstrated that self-efficacy was related to HIV disclosure and QOL, few studies on the mediation role of self-efficacy in the relationship between HIV disclosure and QOL in various domains have been conducted [31, 32].

MSM with HIV/AIDS face the double stigma of having sex with males and their HIV infection status. Compared with promoting disclosure to the general population, disclosing HIV serostatus to male sexual partners was considered a promising behaviour index, which could be changed through behavioural intervention. In this way, we could prevent HIV transmission, improve the QOL of MSM patients and promote a good outcome [33, 34]. Therefore, we conducted this study to explore whether HIV disclosure among MSM patients had an impact on QOL and its various domains, and whether HIV treatment self-efficacy played a mediating role.

## Methods

### Study sites and participants

This study was a secondary analysis of baseline data from a randomized controlled study conducted from October to December 2020 in a hospital in Jinan, Shandong Province, China [35]. The hospital included more than 2000 PLWH, accounting for more than 90% of the PLWH in the city. Among the subjects, MSM patients accounted for approximately 80%. The inclusion criteria for the baseline survey were as follows: (1) 18 years old and above; (2) male sex birth and has had anal sex; (3) HIV-positive; (4) has provided informed consent and volunteered to participate in the study. The exclusion criteria were as follows: (1) inability to complete the survey due to health problems (e.g., mental illness) and (2) inability provide written informed consent.

The original sample size (586) was decided based on the design of a randomized control trial. The sample size for this study was calculated according to the cross-sectional survey formula [36]. The World Health Organization Quality of Life for the HIV-abbreviated version (WHOQOL-HIV BREF) scale is a multidimensional scale, and the mean score of each domain was considered to calculate the sample size. According to previous studies [37], the mean score of MSM with HIV/AIDS in each domain of the QOL was estimated to be 15, the standard deviation was 4, and the relative error was 0.5 [37]. The minimum sample size required was 246, and the existing sample size met the needs of the current research.

### Data collection

MSM with HIV/AIDS were recruited by convenience sampling. The trained medical staff in the hospital referred the research subjects who met the inclusion criteria to the staff of this study. The staff explained the purpose and content of the study to MSM patients who met the inclusion criteria, and distributed paper questionnaires in a relatively closed and independent space of the hospital after obtaining informed consent. After the participants finished the questionnaire, the staff examined and verified the questionnaire on site. Each participant received a compensation of 50 RMB (approximately 7.47 USD). The research protocol was approved by the Ethical Review Committee of the School of Public Health at Shandong University (ethical code: 20190210).

### Measures

#### Socio-demographic characteristics

We collected the socio-demographic characteristics of the participants, including their age, place of residence (1 = urban, 2 = county, 3 = rural areas), job status (1 = stable job or 0 = unstable job), education level (1 = primary school and below, 2 = middle school, 3 = high school or technical school, or 4 = college degree and above) and monthly disposable income (1 = 1500 RMB and below, 2 = 1501–3000 RMB, 3 = 3001–5000 RMB, 4 = 5001–8000 RMB, or 5 = 8001 RMB and above). The response “1500 RMB and below” means very poor, “1500–3000” means low income, “3000–5000” means average income, “5000–8000” means good income, and “8001 RMB and above” means high income.

#### HIV disclosure

We used one question to assess the HIV disclosure status to the male sexual partner of the participants, i.e., “do you inform your male sexual partner of your HIV-positive status?” We dichotomized the responses into “1 = Disclosure” or “0 = Nondisclosure”.

#### HIV treatment self-efficacy

HIV treatment self-efficacy was measured using the revised Chinese version of the AIDS self-efficacy scale (HIV-ASES) [38, 39]. A 10-point Likert scale was used to express patient confidence in their ability to achieve each adherence behaviour. The scale contained 12 items, and its response ranged from 1 = “No confidence at all” to 10 = “Completely confident”, and the total score was between 0 and 120. The higher scores reflected better self-efficacy of MSM patients to HIV treatment adherence. The Cronbach’s alpha coefficient was 0.955 for the scale in this study.

#### Quality of life

It is a multidimensional concept. The Chinese version of the World Health Organization Quality of Life for HIV-abbreviated version (WHOQOL-HIV BREF) scale was used to assess the QOL of MSM patients, which was developed by the WHOQOL-HIV BREF scale in accordance with the Chinese cultural background [40, 41]. The scale contained 31 items that were divided into six domains: physical, psychological, social relationships, independence, environment and spirituality. Each item in each domain had the same weight. The response was a 5-level Likert scale ranging from 1 = “Highly disagree” to 5 = “Highly agree”. The score range of each domain was 4 to 20 and the total score range was 24–120. A higher score indicated a better QOL. The scale had good reliability and validity. The Cronbach’s alpha coefficient was 0.920, and its various domains of Cronbach’s alpha coefficients ranged from 0.586 to 0.821 in this study. Previous studies revealed that the Chinese WHOQOL-HIV BREF scale can be used to measure the QOL of MSM patients in China and to conduct cross-cultural comparative research on QOL [41].

### Statistical analysis

All the data were entered into EpiData 3.1 software by two researchers and tested for consistency. We assessed the normal distribution of continuous variables. Means and standard deviations ($\overline{x }$ ± *s*) or medians (interquartile range, IQR) were used to describe continuous data, and frequencies (percentages) were estimated for categorical variables. We conducted independent-sample *t test*, one-way analysis of variance (ANOVA) and Kruskal–Wallis H tests to assess the differences in QOL and the self-efficacy scores among groups with different socio-demographic characteristics. The variables with *P* < 0.10 in univariate analysis were used as covariates [42].

**Fig. 1:**
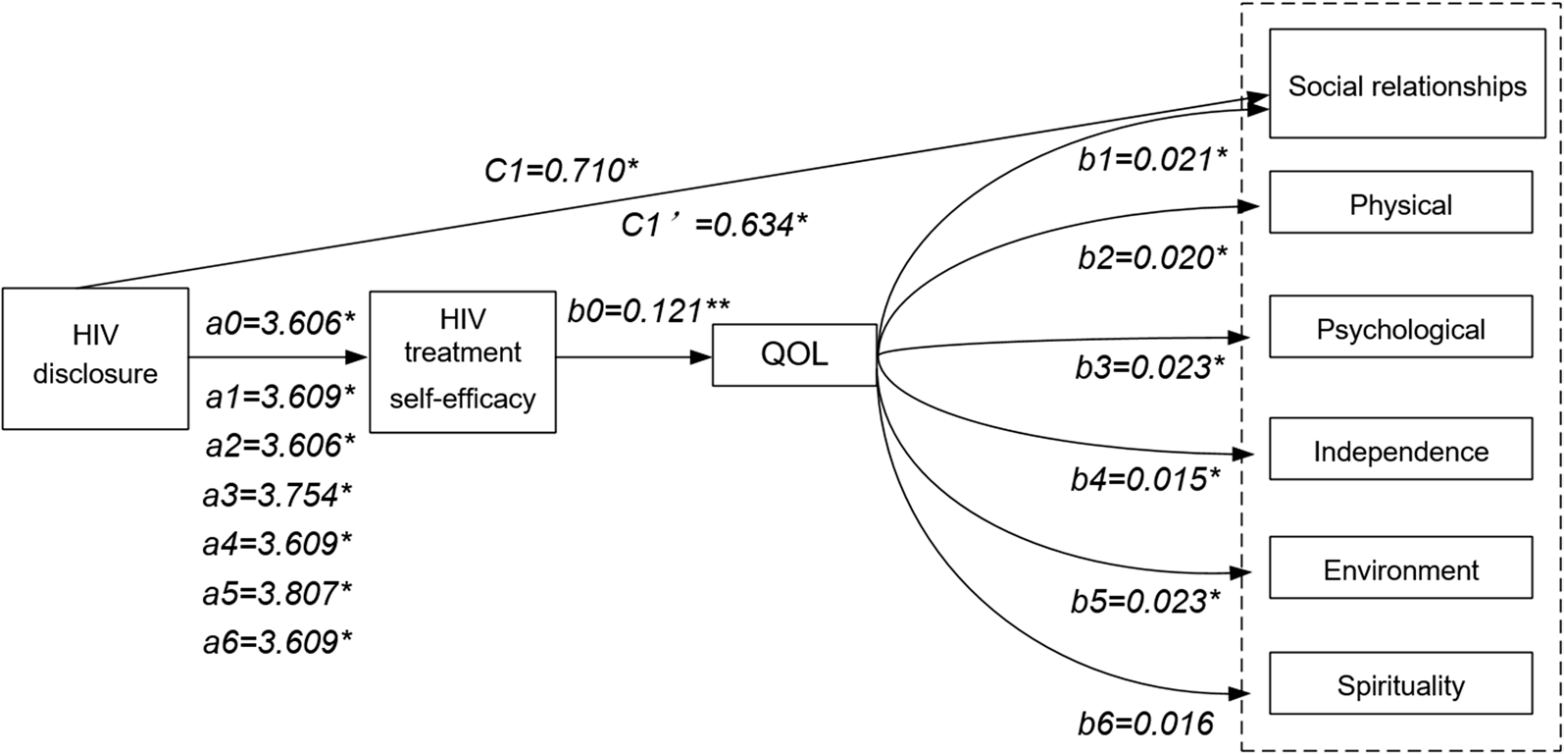
Diagram of mediation effect models of HIV disclosure, self-efficacy and QOL. The variables in the dotted box are the various domains of quality of life. *P < 0.05, **P < 0.001

The mediation analyses were examined using the PROCESS macro for SPSS developed by Hayes (2013) and conducted among every different QOL domain [43]. We constructed mediation analysis models with HIV disclosure as the independent variable (X), HIV treatment self-efficacy as the mediator (M) and QOL and its various domains as the dependent variables (Y). The predictive effect of HIV disclosure on self-efficacy was set as *a*, the predictive effect of self-efficacy on QOL was set as *b*, the total effect of HIV disclosure on QOL was set as *c*, and the direct effect after controlling the mediator was *c*′ (Fig. 1). The bias-corrected nonparametric percentile bootstrap method was used to test the statistical significance of indirect effects based on 5000 resamples. The indirect effects were considered significant if the estimates of the bootstrapped 95% confidence interval (95% *CI*) did not include zero [44]. All the statistical analyses were performed using SPSS version 24.0 and two-tailed *P* < 0.05 was considered statistically significant.

## Results

### Socio-demographic characteristics

A total of 586 MSM with HIV/AIDS were recruited in this study, and 7 participants were excluded due to the large number of logical errors and missing values in their questionnaires. Therefore, a total of 579 participants were included in the analysis. As shown in Table 1, the median age of MSM patients was 33 (28–39), and the age range was wide (age range, 18–82 years). A total of 87.22% of the respondents were 18–44 years old. In addition, 70.98% of them had a stable job, and 61.66% had a college degree or above. Only 4.66% of participants had little disposable income (< 1500RMB), and 16.06% of them disclosed their HIV infection status to their male sexual partners. In all participants, the QOL score was 87.91 ± 12.73, including 14.08 ± 2.75 in the social relationships domain, 14.92 ± 2.49 in the physical domain, 13.88 ± 2.67 in the psychological, 16.84 ± 2.12 in the independence domain, 13.62 ± 2.67 in the environment domain and 14.56 ± 3.55 in the spirituality domain. The median HIV treatment self-efficacy score was 120.00 (116.00–120.00).The QOL scores of MSM who disclosed were higher than those who did not disclose (*t* = − 1.972, *P* < 0.05). In addition, the impacts of having a stable job, the education level, and the monthly disposable income on QOL were statistically significant (t = − 4.473, *P* < 0.001; *H* = 7.855, *P* < 0.001; *H* = 11.397, *P* < 0.001). In six domains of QOL, the scores of MSM patients with stable jobs and high monthly disposable income in all domains of QOL were higher than those without (*P* < 0.05) (as shown in Additional file 1: Table S1).

**Table 1:** Socio-demographic characteristics and relationships with QOL of MSM with HIV/AIDS

Variables	N (%)	QOL^a^	*t*/*H* value	*P* value^c^
Age group			1.964	0.050
18–44	505 (87.22)	88.31 ± 12.81		
45–	74 (12.78)	85.21 ± 11.86		
Registered residence			− 0.316	0.752
Shan Dong Province	471 (81.35)	87.83 ± 12.77		
Other provinces	108 (18.65)	88.26 ± 12.60		
Place of residence			2.096	0.124
Urban	527 (91.02)	88.19 ± 12.84		
Town	24 (4.15)	87.43 ± 12.85		
Rural areas	28 (4.84)	83.16 ± 9.51		
Job status			− 4.473	**<** **0.001**
Unstable	168 (29.02)	84.27 ± 12.61		
Stable	411 (70.98)	89.40 ± 12.49		
Education level^b^			7.855	**<** **0.001**
Primary school and below	3 (0.52)	79.40 ± 5.17		
Middle school	75 (12.95)	81.62 ± 10.84		
High school or technical school	144 (24.87)	88.85 ± 12.56		
College degree or above	357 (61.66)	88.93 ± 12.83		
Monthly disposable income (RMB)^b^			11.397	**<** **0.001**
< 1500	27 (4.66)	85.96 ± 13.12		
1500–3000	68 (11.74)	81.56 ± 12.55		
3001–5000	182 (31.43)	85.70 ± 11.93		
5001–8000	178 (30.74)	89.72 ± 11.89		
8001 and above	124 (21.42)	92.47 ± 13.04		
Ever drinking			− 0.749	0.454
No	244 (42.14)	87.45 ± 12.87		
Yes	335 (57.86)	88.25 ± 12.63		
Ever drugs			− 1.016	0.310
No	546 (93.44)	88.05 ± 12.64		
Yes	38 (6.56)	85.88 ± 13.88		
Marital status with women^b^			0.822	0.482
Single	382 (65.98)	88.50 ± 12.44		
Married	107 (18.48)	86.77 ± 12.93		
Cohabitation	6 (1.04)	85.50 ± 9.90		
Divorced/widowed/separated	84 (14.51)	86.87 ± 13.90		
Having children			− 0.904	0.366
No	439 (75.82)	88.18 ± 12.70		
Yes	140 (24.18)	87.07 ± 12.83		
Medical insurance			1.645	0.100
No	70 (12.09)	85.57 ± 12.83		
Yes	509 (87.91)	88.23 ± 12.70		
HIV disclosure			− 1.972	**0.049**
No	486 (83.94)	87.46 ± 12.74		
Yes	93 (16.06)	90.29 ± 12.46		

See the Additional file 1 for results of the difference between socio-demographic characteristics and other domains of QOL

^a^Mean ± SD. The score of QOL approximately followed the normal distribution

^b^Kruskal–Wallis H test were used to assess the difference of scores of QOL among groups of different socio-demographic characteristics

^c^Bolded P values are statistically significant at *P* < 0.05

### Mediation model analysis

HIV disclosure had a positive effect on self-efficacy, and self-efficacy was then associated with overall QOL and its physical, psychological, independence and environmental domains (*P* < 0.05). However, the effects of HIV disclosure were not significant in these domains after considering self-efficacy.

In the social relationships domain, HIV disclosure significantly predicted HIV treatment self-efficacy (*a*_1_ = 3.609, *P* = 0.035) and HIV treatment self-efficacy, which in turn was associated with better QOL in the social relationships domain (*b*_1_ = 0.021, *P* = 0.004). After the mediator was included, HIV disclosure still had a positive effect on social relationships (${c}^{^{\prime}}$_1_ = 0.634, *P* = 0.035), which indicated that self-efficacy was not the only path between HIV disclosure and the social relationships of MSM patients and played a partial mediation role (Table 2).

**Table 2:** Multivariable analyses with HIV treatment self-efficacy and social relationships as outcomes

Variables	Model 1^a^ (Self-efficacy)				Model 2^b^ (Social relationships)			
	*β*	*se*	*t*	*p*	*β*	*se*	*t*	*p*
Constant	105.600	6.002	17.594	< 0.001	10.077	1.301	7.748	< 0.001
Age group	1.624	2.109	0.770	0.441	− 0.418	0.368	− 1.136	0.257
Registered residence	− 0.932	1.645	− 0.567	0.571	< 0.001	0.287	0.001	1.000
Job status	2.304	1.614	1.427	0.154	0.235	0.282	0.835	0.404
Education level	1.472	0.967	1.523	0.128	0.409	0.169	2.422	0.016
Monthly disposable income	0.455	0.671	0.678	0.498	0.425	0.117	3.629	0.000
Marital status with women	0.822	0.719	1.142	0.254	− 0.200	0.126	− 1.590	0.112
Having children	− 1.238	1.869	− 0.662	0.508	− 0.454	0.326	− 1.391	0.165
HIV disclosure	3.609	1.711	2.109	0.035	0.634	0.300	2.116	0.035
Self-efficacy					0.021	0.007	2.878	0.004
R^2^	0.031				0.103			
F	2.293*				7.242**			

Only the multivariate analysis results with social relationships of QOL as the dependent variable were listed here. See the Additional file 1 for results in other domains of QOL

^a^Self-efficacy was used as outcome variable in Model 1

^b^The score of social relationships of QOL was used as outcome variable in Model 2

^*^P < 0.05, **P < 0.001

In addition, there were no significant effects of HIV disclosure and self-efficacy in the spirituality domain of QOL (${c}^{^{\prime}}$_6_ = 0.400, *P* = 0.088; *b*_6_ = 0.010, *P* = 0.093). Detailed multivariable analysis results for each domain of QOL were provided in Additional file 1: Tables S2–S7.

### Mediation effects testing

The bias-corrected nonparametric percentile bootstrap method was conducted to assess the mediation effects with 5000 resamples. As shown in Table 3, the indirect effects through self-efficacy were all statistically significant (95% CI did not include zero) on the overall QOL and its various domains, which means that self-efficacy was the potential mechanism for explaining the relationship between HIV disclosure and QOL. The mediation effect accounts for 10.70% of the total effect on the domain of social relationships. However, the confidence interval of the mediation effect in the spirituality domain was very close to 0, and the regression coefficient was not statistically significant. It was believed that the mediation effect was very weak and can be ignored. The diagram of the mediation effect model was shown in Fig. 1.

**Table 3:** Indirect effects testing of mediation analyses

Variable	Indirect effect	SE	Boot 95%CI	
			LLCI	ULCI
QOL	0.436	0.148	0.183	0.763
Social relationships	0.076	0.027	0.029	0.133
Physiology	0.071	0.029	0.020	0.135
Psychology	0.089	0.032	0.033	0.158
Independence	0.056	0.023	0.018	0.107
Environment	0.086	0.032	0.032	0.155
Spirituality	0.059	0.035	0.001	0.137

*LLCI* lower limit 95% confidence interval, *ULCI* upper limit 95% confidence interval

## Discussion

In this study we explored the mediating role of HIV treatment self-efficacy between HIV disclosure and QOL among MSM with HIV/AIDS. The findings demonstrated that HIV disclosure was positively associated with self-efficacy, which was related to a better QOL, including the physical, psychological, social relationships, independence and environment domains. The mediation analyses indicated that self-efficacy played an important role in the relationship between HIV disclosure and QOL [25, 45–47]. Consistent with the results of a meta-analysis, the proportion of individuals disclosing their HIV infection status to sexual partners was very low (12–53%) and varied according to personal and situational factors [48]. For most MSM patients, it is a great challenge to disclose their HIV infection status. Our study showed that only 16.06% of participants disclosed their HIV infection status to their male sexual partners. Disclosure of HIV infection status is clearly related to reducing HIV transmission, promoting treatment adherence and promting good health [8]. In this study, the QOL scores and its domains of participants with HIV disclosure were higher than those who did not disclose. Although the differences in some domains were not statistically significant, it also showed that HIV disclosure has a positive impact on most domains of QOL. Unlike the general disclosure of previous studies, HIV disclosure makes it easier to obtain understanding, emotional support and positive response. It will increase MSM patients’ belief in fighting disease, improve their self-efficacy, help them actively seek medical treatment strategies, and reduce the probability of missing drugs or hindering their access to medical services [49, 50]. In addition, disclosure behaviour is a positive and reinforcing experience that helps patients accept their disease condition. The encouragement of male sexual partners is combined with their own expectations, which enhances their treatment motivation and confidence. Improving self-efficacy in turn can reduce the expected pressure and promote mental health while improving social relationships. This finding is aligned with the results that the positive rate of depression and other negative results of those who refuse to disclose their HIV status were higher than those who disclose their HIV status [51]. HIV disclosure is an important contributing factor to mental health. Moreover, disclosure behaviour is affected by the surroundings but also affects the surrounding people and environment. We found that disclosure was an important predictor in domains of good social relationships and environment, which was consistent with previous results [26]. We found that disclosure did not have a significant impact on the spirituality domain, either as a direct effect or an indirect effect, which was consistent with a previous study [6].

Our study revealed the mediation mechanism that explained the impact of HIV disclosure on the QOL of MSM with HIV/AIDS. The results showed that self-efficacy was an important mediator that was related to the overall QOL and its physical, psychological, social relationships, independence and environment domains. Self-efficacy was considered to be the key determinant of health promotion behaviour based on social cognitive theory (SCT) [52]. As noted in the study by Jiang, MSM patients with higher self-efficacy were usually more able to accept the reality of the illness and their own health status, had a better mentality and positively sought medical treatment [30]. Therefore, self-efficacy can not only improve their physical health and enhance their autonomy but also enable them to access a good psychological state by reducing anxiety and depression. Previous studies in China also have shown that self-efficacy can help MSM patients make use of various resources to manage disease conditions and reduce the negative impact on their health [31, 53]. Self-efficacy has been shown to be a predictor of health behaviours, such as promoting treatment adherence, and it plays an important role in improving the QOL of MSM patients [54].

This study has some limitations. First, it was a cross-sectional study, which limited the ability to infer the causal and temporal relationships between variables. Future studies should use longitudinal and intervention designs to validate our findings and explore the long-term outcomes of disclosure. Second, the participants were recruited through convenience sampling, which may not fully represent all MSM patients, and selection bias cannot be excluded. Third, self-report measurement may induce social desirability bias, and future research must evaluate the research variables more objectively. Despite these limitations, our results increase the current understanding of the potential path of HIV on QOL and its impact on various domains in MSM patients.

## Conclusions

MSM living with HIV/AIDS who disclosed their HIV status to male sexual partners were more likely to have a good QOL. This study showed that HIV disclosure not only had a positive direct effect on the social relationships domain of QOL but also had an indirect impact on it through self-efficacy. In the overall QOL, physical, psychological, independence and environment domains, it only had an indirect impact through self-efficacy. The results emphasized the importance of HIV disclosure for QOL among MSM patients and suggested that health care providers should assist MSM infected persons in deciding whether to disclose their HIV infectious status during daily medical services. In addition, it is not enough to promote disclosure to male sexual partners but also to increase the intervention related to HIV treatment self-efficacy, such as cognitive behavioural interventions [55], to improve the health-related quality of life of MSM with HIV/AIDS.

## Supplementary Information

**Additional file 1: Table S1.** Socio-demographic characteristics and relationships with QOL and its various domains a (n = 579). **Table S2.** Multivariable analyses with HIV treatment self-efficacy and overall QOL as outcomes. **Table S3.** Multivariable analyses with HIV treatment self-efficacy and physical as outcomes. **Table S4.** Multivariable analyses with HIV treatment self-efficacy and psychological as outcomes. **Table S5.** Multivariable analyses with HIV treatment self-efficacy and independence as outcomes. **Table S6.** Multivariable analyses with HIV treatment self-efficacy and environment as outcomes. **Table S7.** Multivariable analyses with HIV treatment self-efficacy and spirituality as outcomes.

## Data Availability

The datasets generated during the current study are not publicly available due to privacy or ethical restrictions but are available from the corresponding author on reasonable request.

## Abbreviations

AIDS: Acquired immune deficiency syndrome
HIV: Human immunodeficiency virus
ART: Antiretroviral therapy
PLWH: People living with HIV
QOL: Quality of life
MSM: Men who have sex with men
UAI: Unprotected anal intercourse
SCT: Social cognitive theory
HIV-ASES: AIDS self-efficacy scale
WHOQOL-HIV BREF: World Health Organization Quality of Life for HIV-abbreviated version
IQR: Interquartile range
ANOVA: One-way analysis of variance
CI: Confidence interval
